# Slaughter House Examinations

**Published:** 1882-09

**Authors:** 


### SLAUGHTER-HOUSE EXAMINATIONS.

The importance of facts to be obtained by a careful and systematic examination of animals slaughtered for food has hardly been recognized in this country. Reports of such examinations made in other countries have frequently been found to contain most valuable information. In particular, the city of Augsburg may be instanced. For some years the investigations of Inspector Adam have been widely quoted by Fleming and others. Recently the *Journal of Comparative Medicine* gives some important data obtained from the same source. Among 66,731 animals brought to market in the year 1881, there were 12,269 cattle, of which 246, or 2.01 per cent., were found tuberculous. This ratio is much smaller than is generally supposed to exist among American cattle. But further, the amount of tuberculosis varied greatly,and in most cases was not extensive enough to injure seriously the quality of the meat. In fact, only eighteen animals out of the whole two hundred and forty-six were condemned as unfit for food. The tubercles were confined to the lungs in 142 cases, to the pleura in 37 cases, to the lungs and pleura in 68 cases. In 59 cases there were also tubercles in the liver.

Thus the important fact was brought out that in no case were tubercles found in parts of the body (except the liver) used as food.

Further facts bearing upon the infectiousness of the milk of tuberculous cows are given. Among 24,901 calves killed, when under the age of four weeks, no tubercles were found. Now, about five hundred of these must have been sucking the milk of tuberculous mothers, yet apparently with no injurious effect. And furthermore, the age of the tuberculous cattle was, with the exception of twenty-three, over three years. The conclusion is, that cattle, at least, are not infected, in the ordinary sense, with tnberculosis by feeding upon the milk from diseased mothers.—*Medical Record.*

